# A dataset for deep learning based detection of printed circuit board surface defect

**DOI:** 10.1038/s41597-024-03656-8

**Published:** 2024-07-22

**Authors:** Shengping Lv, Bin Ouyang, Zhihua Deng, Tairan Liang, Shixin Jiang, Kaibin Zhang, Jianyu Chen, Zhuohui Li

**Affiliations:** 1 School of Engineering, South China Agricultural University, No. 483, Wushan Road, Guangzhou, 510642 China; 2 Guangzhou FastPrint Technology Co., Ltd, No. 33, Guangpuzhong Road, Guangzhou, China; 3 CEPREI, No. 78, Zhucun Avenue West, Guangzhou, 511370 China

**Keywords:** Computer science, Electrical and electronic engineering

## Abstract

Printed circuit board (PCB) may display diverse surface defects in manufacturing. These defects not only influence aesthetics but can also affect the performance of the PCB and potentially damage the entire board. Thus, achieving efficient and highly accurate detection of PCB surface defects is fundamental for quality control in fabrication. The rapidly advancing deep learning (DL) technology holds promising prospects for providing accurate and efficient detection methods for surface defects on PCB. To facilitate DL model training, it is imperative to compile a comprehensive dataset encompassing diverse surface defect types found on PCB at a significant scale. This work categorized PCB surface defects into 9 distinct categories based on factors such as their causes, locations, and morphologies and developed a dataset of PCB surface defect (DsPCBSD+). In DsPCBSD+, a total of 20,276 defects were annotated manually by bounding boxes on the 10,259 images. This openly accessible dataset is aimed accelerating and promoting further researches and advancements in the field of DL-based detection of PCB surface defect.

## Background & Summary

The printed circuit board (PCB) plays a vital role in electronic devices, serving the dual purposes of mechanically supporting and establishing electrical connections among diverse electronic components^1^. PCB finds applications in virtually all types of electronic information devices, spanning from 3C (computer, communication, and consumer) devices, household appliances, automobile electronics and more^2^. The quality of PCB is crucial for the overall performance of electronic equipment. Consequently, PCB manufacturers are expected to provide products with high quality, high precision, and high reliability. Therefore, implementing rigorous quality control measures throughout the fabrication process and effectively detecting defects on PCB is of paramount importance. If recurring defects cannot be promptly and precisely detected, it is highly likely that many manufactured PCB will eventually be scrapped, resulting in both wastage and incurring significant costs^3^.

**Fig. 1:**
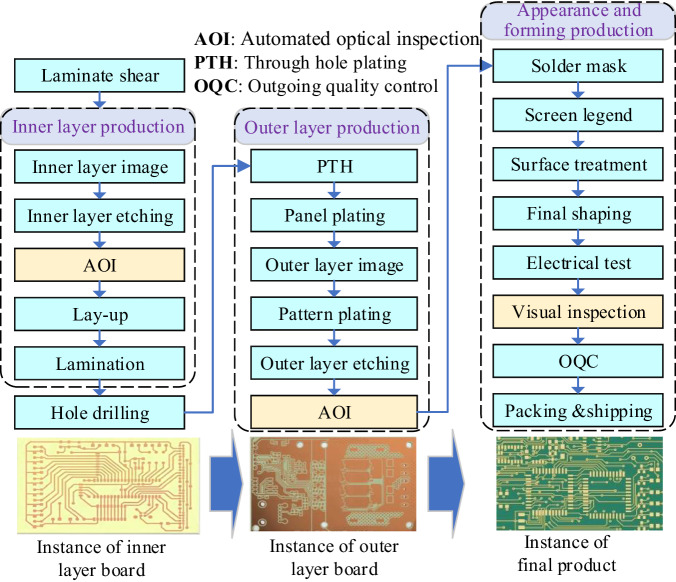
Overview of fabrication process for multilayer PCB.

The PCB fabrication process is complex, particularly for multilayer boards. Figure 1 illustrates the fabrication process of multilayer PCB. Due to factors such as technical faults, polluted work environment, device anomalies, and manual mishandling, various surface defects are inevitably introduced at different stages^4^. These defects encompass issues such as open, short, spur, spurious copper, foreign object, and more, affecting various element composition of PCB, including the copper wire, copper surface, hole, base material, and so forth. These surface defects not only affect the aesthetics but can also significantly impair the performance of PCB or even cause extensive damage to the entire board. Therefore, the PCB workshop explicitly mandates defect inspections for each inner/outer layer board after etching, as well as before shipping. Efficient and accurate detection of these various defects is crucial to ensure quality in the PCB fabrication.

Previously, surface defect detection of PCB was typically carried out through manual visual inspection. However, manual visual suffers from drawbacks such as heavily relying on experienced inspectors, significant subjectivity, high labor intensity, low consistency and efficiency^5^. As the demands for precise and efficient inspection of PCB increase, especially with the trend towards greater complexity, tiny, and intricacy, manual inspection is gradually being phased out. In the past decades, there has been a heightened emphasis on utilizing automated optical inspection (AOI) for defect detection in the PCB production, and empirical evidence suggests that it has significantly enhanced both the detection accuracy and efficiency^2,3^.

The detection algorithm, which serves as the core component of AOI software, falls into three categories: traditional image processing approaches, machine learning approaches, and deep learning (DL)-based approaches. Traditional image processing approaches can still be classified into three types: referential methods, non-referential methods, and hybrid methods–which involve a combination of more than one of these methods^5^. The referential methods compare images or extracted features of the inspected PCB with predefined template or features to identify the locations of defects^6,7^. Referential methods have been widely used but suffer from several drawbacks^3^, including heavy dependence on the quality of templates, the need for additional preprocessing, calibration, and post-processing, and time-consuming pixel-level comparison matching and so on. Non-referential methods recognize defects based on pre-designed rules or by assuming that features are simple geometric shapes, where defects manifest as unexpected irregular features. However, these methods might overlook significant flaws and distorted features^3^.

Machine learning-based approaches such as decision trees^8^ and support vector machines^9^ have also been integrated into AOI to enhance detection accuracy. However, these approaches share the same issues as traditional image processing approaches^3^. Additionally, machine learning approaches require the configuration of various parameters and often necessitate additional preprocessing and post-processing for defect image handling. Based on the aforementioned achievements, the existing AOI detection accuracy, speed, and level of automation have greatly improved compared to manual visual inspection. However, practical experience has revealed that AOI equipment still has a high likelihood of falsely identifying or misjudging defects, necessitating the involvement of a significant number of specialized personnel for time-consuming visual rechecks.

With the rapid advancement of DL technology in recent years, there has been an increasing emphasis on end-to-end DL-based approaches in AOI detection algorithm research. The goal is to improve both efficiency and detection accuracy^10,11^ while addressing the limitations associated with traditional image processing and machine learning-based approaches. To effectively support the training of DL models, there is a need to construct a dataset that comprehensively covers various types of PCB surface defects and has a significant scale.

In recent years, several publicly available PCB defect datasets have been created for training and evaluating DL models. Tang *et al*.^1^ constructed a dataset called DeepPCB, which can be accessed at https://github.com/tangsanli5201/DeepPCB. This dataset consists of 1,500 pairs of defect images (template and tested images) covering 6 common types of surface defects, including open, short, mousebite, spur, copper, and pinhole. All images in the DeepPCB dataset were captured using a linear scan charge coupled device. Additionally, a number of artificial defects were manually introduced into each test image, resulting in approximately 3 to 12 defects per image. The MeiweiPCB dataset^12^, available at https://github.com/youtang1993/MeiweiPCB), comprises 939 defect images randomly cropped from original images acquired using an industrial line scan camera. Defects in MeiweiPCB were not categorized, and labels for these defects were provided in two forms: pixel-wise mask annotation and bounding box annotation. Huang *et al*.^13^ published a PKU-Market-PCB dataset, which contains 1,386 defect images categorized into 6 categories: missing hole, mouse bite, open, short, spur, and spurious copper. The dataset can be accessible at https://robotics.pkusz.edu.cn/resources/dataset/. Out of these images, 693 originated from 10 PCB, while the remaining 693 images were generated through rotation augmentation. The PKU-Market-PCB dataset has been extensively utilized to validate the performance of DL models^13–16^, and has been augmented by some researches^15,16^. Ding *et al*.^15^ expanded the dataset to 10,668 images using geometric and image transformations. Du *et al*.^16^ constructed a dataset comprising 693 images of normally placed and 507 images randomly rotated based on PKU-Market-PCB.

Researchers have also developed some unpublicized datasets. Hu *et al*.^17^ constructed a dataset comprising 1,500 defect images, covering 6 common types of defects: open, short, mouse bite, spur, pinhole, and solder ball. All the images in the dataset were cropped from the original images captured using a camera. Rotation and brightness adjustment were introduced to the dataset to augment the original images, resulting in a total of 12,000 defect images. Liao *et al*.^18^ developed a dataset comprising 19,029 defect images. Each image encompasses one of the following surface defects: bumpy, clutter, scratch, line repair damage, hole loss, or over oil-filling. Among these, 2,008 images were randomly cropped from original images acquired using an industrial camera. The other 17,021 were generated using augmentation techniques, such as random rotation, cropping, translation, horizontal and vertical flipping, and luminance balancing. Adibhatla *et al*.^19^ extracted 11,000 images from the AOI machine to compile a PCB dataset. The dataset comprises images with 11 types of defects; however, all the defects were labeled as a single defect type. Adibhatla *et al*.^20^ built a dataset comprising 23,000 PCB surface defect images. All the defect images were collected from an automated visual inspection (AVI) machine. Each defective region in the image was labeled as DEFECT with bounding box. Pham *et al*.^21^ created a dataset consisting of 22,909 surface defect images. The original PCB images were obtained through an AFVI system. Subsequently, defects were extracted and saved as cropped images, with the defects positioned at the center of each image. These defect images were classified as either true or false defects. Zhang *et al*.^11^ provided a PCB-2 dataset consisting of 40,706 images and two classes of defects: real defect and pseudo defect. Li *et al*.^22^ constructed a dataset containing 2,000 images and five categories of defects, including copper short, short, open, near open and near short. Each category has 400 defect images.

The aforementioned datasets support the training of DL models and accelerate the research on DL-based algorithms. However, the currently constructed datasets have several shortcomings.

(1) PKU-Market-PCB^13^ and its extended versions^15,16^, as well as DeepPCB^1^ are primarily generated through artificial synthesis. The majority of defects in the datasets constructed by Ding *et al*.^15^, Hu *et al*.^17^ and Liao *et al*.^18^ were generated by augmenting a small set of original defects. This synthesis or augmentation has led to very limited intra-class variability and has created significant disparities from real defects that occur in the PCB production process.

(2) The defects in certain datasets lack categorization^12,19–21^ or they were roughly divided into two categories^11^. Meanwhile, some datasets encompass five^22^ or six^1,13–18^ defect categories but have a limited number of defect samples, with a maximum of 2,008 defects. Therefore, there is a need to enhance the coverage of surface defects on PCB and refine the categorization.

(3) The division of training and validation sets in some datasets^1,13– 18^ was conducted after augmenting the original defects. This results in the validation set containing a significant number of defect samples that are highly similar to those in the training set, making it challenging to validate the detection accuracy and generalization performance of DL models for real-world applications.

(4) The AOI/AVI system often crops multiple defect images using a sliding window, and these cropped images may display defect-free instances, duplicate defects, incomplete defects, and so on. However, datasets sourced from AOI/AVI^11,19,20,22^ do not provide details on how to address this situation. Additionally, there is no guidance on handling defects that cannot be determined solely through visual inspection and highly unbalance among different categories of defects. Furthermore, these datasets have not been publicly shared.

To overcome these limitations, the goal of this study is to establish a publicly available dataset that focuses on the surface defects of inner and outer layer boards of PCB, covering multiple defect categories. The dataset, named DsPCBSD+ (Dataset of PCB surface defect), comprises images sourced from actual PCB produced at Guangzhou FastPrint Technology Co., Ltd. Experts in PCB have meticulously classified defects into 4 primary categories considering their causes: copper residue, copper deficiency, conductor scratch, and foreign object. These 4 categories are further subdivided into 9 categories considering factors such as locations and morphologies. The 9 defect categories comprise Short (SH), Spur (SP), Spurious copper (SC), Open (OP), Mouse bite (MB), Hole breakout (HB), Conductor scratch (CS), Conductor foreign object (CFO), and Base material foreign object (BMFO). In total, this dataset comprises 10,259 defect images and 20,276 manually annotated defect bounding boxes. This openly accessible dataset aims at accelerating and promoting further research and advancements in the field of intelligent detection of PCB defects.

## Methods

**Fig. 2:**
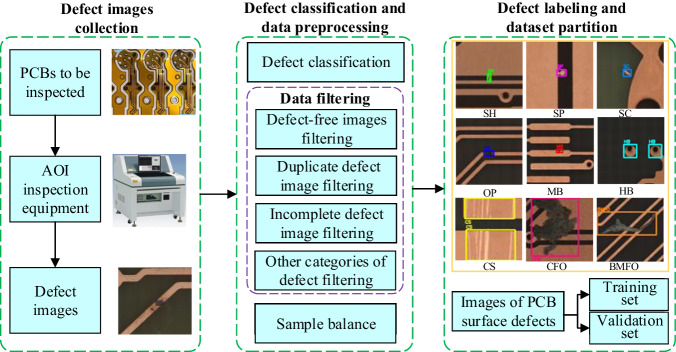
Overview of the construction process for DsPCBSD+.

The construction procedure for DsPCBSD+ is illustrated in Fig. 2 and is primarily divided into the following three steps: (1) Defect images collection; (2) Defect classification and data preprocessing; (3) Defect labeling and dataset partition.

### Defect images collection

This study exclusively utilized images of actual PCB defects from the inner and outer layers of boards after etching, gathered from the AOI equipment AGLE’OL AOI-100 V8 in the workshop of Guangzhou FastPrint Technology Co., Ltd. These two equipment employ the multi-group of controllable LED spotlight system for illumination, coupled with a 16K high-resolution line scan system for image acquisition. The line scan image acquisition system comprises four cameras mounted on the top and an additional four on the bottom. These cameras are utilized to capture images of both sides of the PCB. When capturing PCB images, the linear sensor scans line by line along the horizontal direction of the PCB under inspection. Image is captured and transmitted in digital form to the image processing unit. In the image processing unit, the captured images undergo preprocessing, which includes tasks such as noise removal, contrast enhancement, and brightness adjustment. Subsequently, key features are extracted from these images based on element feature learning and sub-pixel contour comparison approaches. The processor meticulously compares these extracted features with predefined reference images to identify any potential defects and their locations. All defect images are systematically archived in a management system, enabling comprehensive analysis, traceability, and continuous quality enhancement. A total of 32,259 images have been directly retrieved from the management system to compose the DsPCBSD+ dataset. Each image is formatted in JPG and has dimensions of 226 × 226 pixels.

### Defect classification and data preprocessing

PCB surface defects are diverse and have traditionally been classified based on the understanding of personnel within PCB manufacturing facilities, rather than being categorized according to the requirements of DL-based detection. In this study, features of PCB surface defects are abstracted based on their causes, locations and morphologies, and defect types are redefined here.

**Fig. 3:**
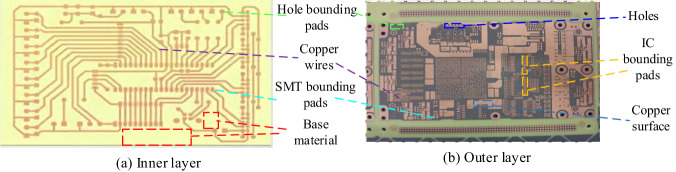
Schematic diagram of element compositions on inner/outer layer.

**Fig. 4:**
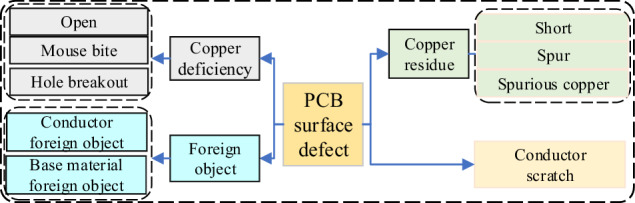
PCB surface defect classification.

Considering the causes, PCB surface defects are primarily influenced by factors such as copper residue, copper deficiency, conductor scratches, and foreign objects. Consequently, these defects have been classified into 4 distinct categories: Copper residue defect, Copper deficiency defect, Conductor scratch defect, and Foreign object defect. In terms of the locations where these surface defects manifest, they can occur across various elements and compositions on the PCB. Figure 3 provides a visual representation of the principal element compositions for inner and outer layer boards. Inner layer boards primarily consist of wires, hole bounding pads, surface mount technology (SMT) bounding pads, and the base material area. On the outer layer boards, the main element compositions encompass copper wires, copper surfaces, holes, and pads (including hole bounding pads, SMT bounding pads, and integrated circuit (IC) bounding pads). IC/SMT/hole bounding pads can be regarded as relatively small areas of copper surfaces. And copper wires, copper surfaces, IC/hole/SMT bonding pads can be collectively referred to as conductors. In summary, the locations on PCB where surface defects occur can be categorized into three main groups: conductors, holes, and the base material. Subsequently, these defects are further subdivided into 9 categories based on their locations and morphologies, as depicted in Fig. 4.

A Short on a PCB refers to an unintended connection between two or more distinct conductors resulting from the presence of residual copper. Short defects primarily occur between different wires, and they can also occur between copper surfaces and wires, or between copper surfaces. Spur defects are identified by irregular protrusions along the edges of conductors, often manifesting as sharp, pointed shapes. The Spur defect may lead to incorrect connections between conductors, potentially causing short or disrupting the normal operation of the circuit. Spurious copper refers to irregular or unwanted copper residue found on the base material, within holes, or on copper surfaces. The presence of this unintended copper material can result in incorrect circuit connections, short circuits, and other potential circuit issues.

An Open defect on PCB occurs when the connection path within the conductor is interrupted, hindering the flow of electric current along that specific pathway. A Mouse bite on a PCB refers to small, localized depression or crack that appear at the edge of conductors. It may result in poor connections in the circuit or connections that are close to being interrupted. The Hole breakout typically refers to a situation where the center of a hole significantly deviates from the hole bounding pad, resulting in a noticeable lack of copper material along some edges of the hole. Hole breakout affects the functionality and performance of the holes, especially in multi-layer PCB where holes are used to connect different layers of circuits.

Defects on copper wires or copper surfaces stemming from scratches, appearing as linear or multiple linear defects, fall under the category of Conductor scratch. Such scratches on elements like wires and pads can result in suboptimal or interrupted electrical connections, impacting the overall functionality of the device. Concurrently, these scratches may undermine the mechanical strength between components, making the PCB more vulnerable to the effects of vibrations, impacts, or temperature fluctuations, ultimately diminishing the device’s reliability.

The morphological characteristics of foreign objects appearing on conductors or the base material vary significantly. Impurities, bubbles, dirt, deposits, or other substances on the conductors are categorized as Conductor foreign object. Meanwhile, the unintentional presence of contamination such as oils, oxides, chemical etching, cleaning agents, solvents, fingerprints, stains and so forth on conductors is also classified as Conductor foreign object. When foreign materials, such as bubbles, chips, particles, or other substances, appear on the base material, they are classified as Base material foreign object. Similarly, contamination, such as grease, adhesives, oxidation, corrosion, and other forms of pollution, visually resembling foreign objects to a significant extent, is also categorized as the defect type of Base material foreign object. The background of holes is similar to base material, and the foreign objects within the holes generally exhibit morphological characteristics similar to those of Base material foreign object. Consequently, when foreign objects are present inside the holes, they are categorized as Base material foreign object. Foreign objects on a PCB have the potential to result in short circuits between components that should not be connected, obstruct connections between circuit elements, or interfere with circuit signals. These issues ultimately lead to reduced device performance.

During the dataset construction process, all the source images potentially containing abnormal data should be systematically eliminated, either manually or automatically. This includes defect-free images, duplicate defect images, incomplete defect images, and other categories of defect images in the original dataset.

Defect-free images represent cases where no defects are present or the defects are too subtle to be discerned by the naked eye. The defect-free images are manually screened and excluded. AOI often captures multiple photos of the same defect to enhance the accuracy of detecting and confirming potential defects. Consequently, this leads to the storage of duplicate defect images with partially overlapping regions. In such cases, the hash value matching method is employed to compare all images and one of images is retained. Subsequently, additional manual screening is conducted, and the image with the highest defect percentage is retained for dataset construction. The incomplete defect image signifies an image containing defects that require clear boundaries, but some boundaries related to the defects are missing in the image. For example, the image with Open object only includes one end of the conductor for this defect, while the image with Short object only contains one side of the conductor for this defect. Other categories of defect images involves images that do not include any of the aforementioned 9 specific defect categories, such as exceeding tolerances in conductor width/spacing, depressions in blind vias, or missing drilled holes. These defects are determined through template matching but cannot be identified solely through 2D visual inspection. Other categories of defect images are manually excluded from the source images.

After the aforementioned screening, the retained images exhibit a notable imbalance among the 9 types of defect objects. Specifically, there is a few of OP and SH defects, while a substantial number of CFO and BMFO defects are present. Training DL models on such imbalanced samples can introduce bias in feature learning, leading the model to prioritize better recognition of categories CFO and BMFO with more samples, resulting in suboptimal performance on OP and SH with fewer samples. Given that OP and SH defects can directly lead to the scrapping of PCB, whereas CFO and BMFO defects typically do not directly lead to PCB scrapping, it is crucial to ensure that the model avoids biasing towards learning features of CFO and BMFO at the expense of recognizing OP and SH defects. To address this issue, all images containing Open and Short defects are included in the DsPCBSD+ dataset. As for defects in other categories, an initial subset of images is randomly selected for defect labeling, and additional images are included based on the statistical results derived from labelled bounding boxes for various defect categories. This process endeavors to establish a more balanced distribution of defect types, thereby ensuring a more robust and equitable performance across all defect categories.

### Defect labeling and dataset partition

**Fig. 5:**
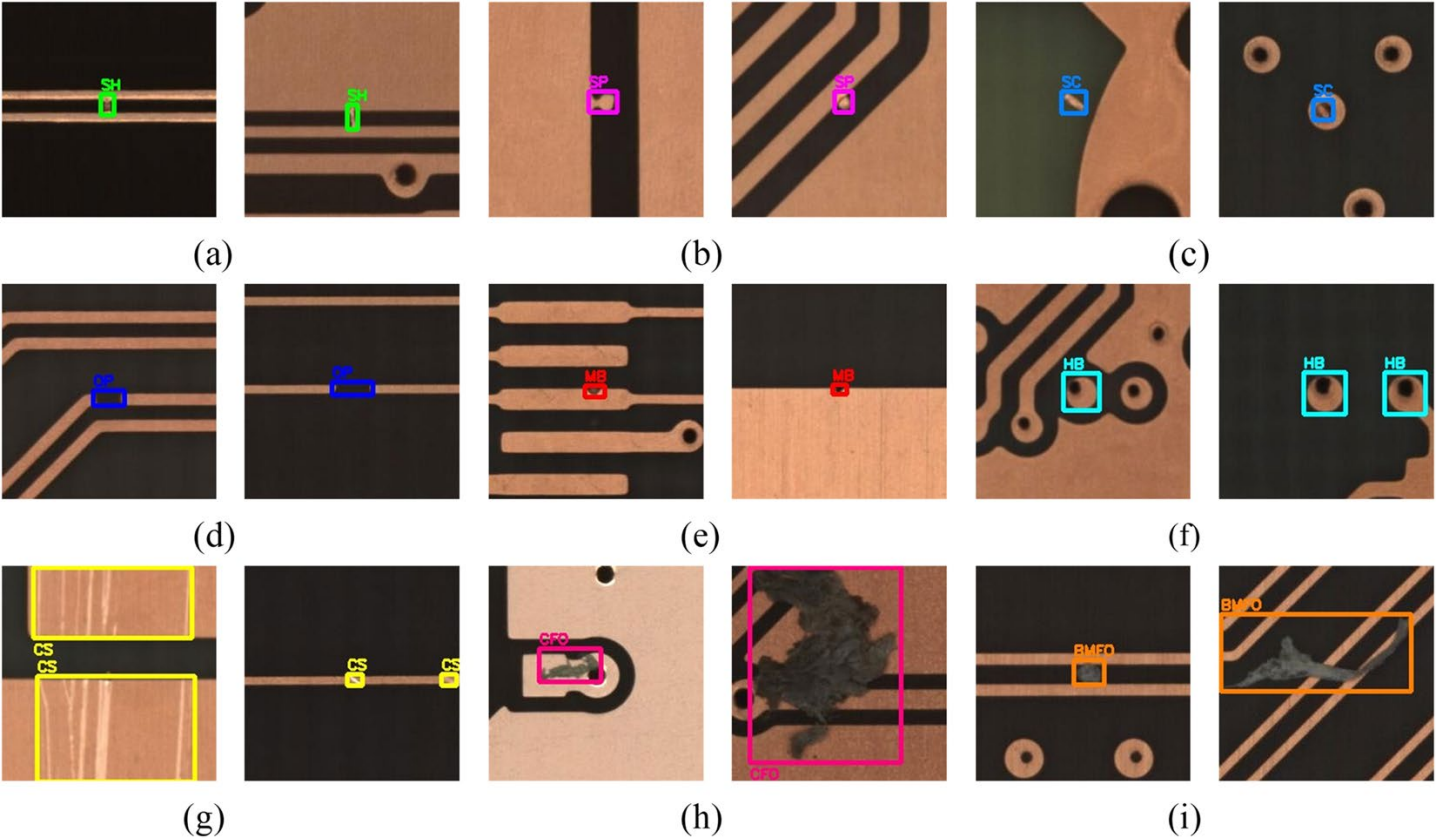
Annotation instances of the 9 categories of defect. (**a**) SH; (**b**) SP; (**c**) SC; (**d**) OP; (**e**) MB; (**f**) HB; (**g**) CS; (**h**) CFO; (**i**) BMFO.

**Fig. 6:**
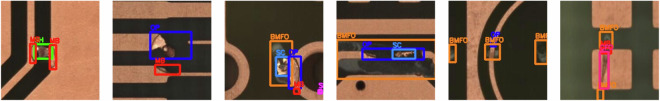
Annotation instances of multi categories of defect on one image.

Finally, the categorized defects were annotated and the labels were named using LabelImg software. An official open-source download link for the LabelImg software is provided here: https://github.com/heartexlabs/labelImg. Researchers have the flexibility to assign labels in YOLO, VOC, or CreateML format and annotate the images following the guidelines provided by the official documentation. The YOLO, VOC, or CreateML formats can be mutually converted. In this study, labels were initially formatted in the VOC style before annotation. For each defective image, a bounding box was meticulously drawn around the respective defect. Subsequently, the bounding box was labeled with abbreviations of defect categories, including SH (Short), SP (Spur), SC (Spurious copper), OP (Open), MB (Mouse bite), HB (Hole breakout), CS (Conductor scratch), CFO (Conductor foreign object), and BMFO (Base material foreign object). The annotation instances for the 9 categories of defects were presented in Fig. 5. In practical production, various defects of different shapes and sizes often occur on the surface of PCB. These defects may simultaneously affect multiple element compositions of the PCB, such as copper wires, copper surfaces, holes, and base materials, ultimately resulting in multiple categories of defect on one image. In such scenarios, each type of defect had to be individually annotated, and the annotation instances are depicted in Fig. 6.

**Fig. 7:**
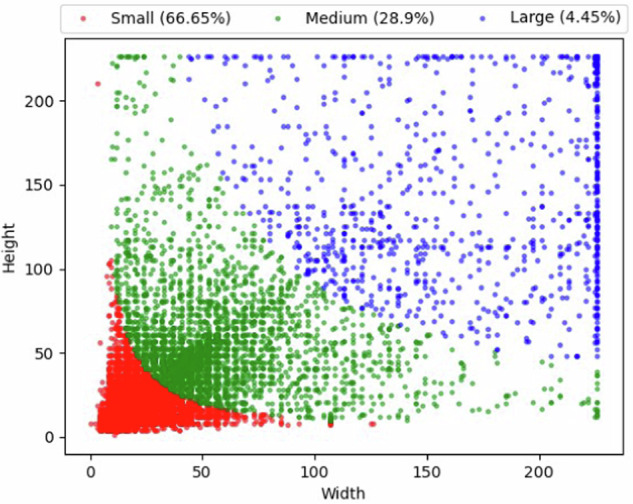
Size distribution of defect bounding boxes in DsPCBSD+.

After completing the annotation, we obtained a collection of images with labeled bounding boxes and VOC-structured XML files containing information about these bounding boxes and labels. Finally, a total of 20,276 defects were annotated by bounding boxes on the 10,259 images. The VOC datasets were then converted into YOLO and COCO datasets through script files and stored. According to the definition of COCO^23^, objects with area of ground truth less than 32 × 32 pixels, between 32 × 32 pixels and 96 × 96 pixels and larger than 96 × 96 pixels are taken as small, middle and large-sized objects, respectively. The size distribution of objects is shown in Fig. 7. In addition, the distribution of three types defect labels across different categories in DsPCBSD+ has been compiled and given in Table 1. Notably, there is a significant variation in the size of defects within DsPCBSD+. It can also be seen that Short, Spur, Spurious copper, Open and Mouse bite exhibit relatively small differences in size, while others, like Conductor scratch, Conductor foreign object, and Base material foreign object show significant variations in size. By combining the defect instances given in Fig. 5, it is evident that the defect categories with significant size variations also exhibit substantial intra-class differences.

**Table 1:** Count of three size labels.

Categories	Small	Medium	Large	All
Short (SH)	710	205	0	915
Spur (SP)	4469	115	0	4584
Spurious copper (SC)	1352	231	10	1593
Open (OP)	1406	361	3	1770
Mouse bite (MB)	2421	108	0	2529
Hole breakout (HB)	35	2848	0	2883
Conductor scratch (CS)	734	1043	713	2490
Conductor foreign object (CFO)	1140	582	110	1832
Base material foreign object (BMFO)	1308	304	68	1680
Total	13575	5797	904	20276

**Fig. 8:**
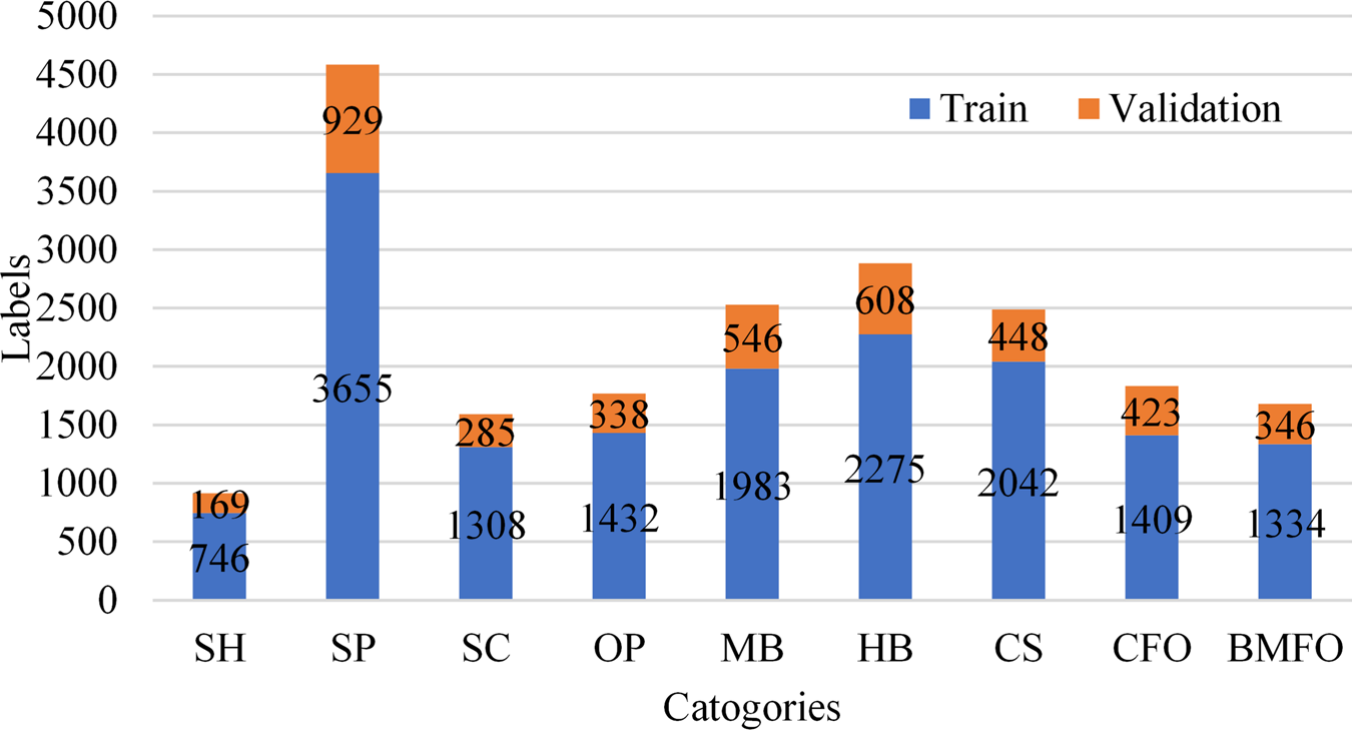
Distribution of defect labels in the training and validation sets across different categories.

Based on the annotated dataset, images were randomly divided into training and validation sets at an 8:2 ratio. There are 8,208 images in the training set and 2,051 images in the validation set. The corresponding labels are 16,184 and 4,092 for the training and validation sets, respectively. Figure 8 provides the number of labels for the 9 categories of defects in both the training and validation sets.

## Data Records

The DsPCBSD+ are freely available in the Figshare repository^24^. Due to their attributes of simplicity, flexibility, and universality, YOLO and COCO dataset formats have garnered popularity in both academic and industry. Consequently, data annotations in this study are provided in YOLO and COCO formats, with images and annotation files stored separately in the Data_YOLO and Data_COCO folders.

There are two subfolders under Data_YOLO: images and labels, in which respectively stores image data and label data. Within these subfolders, there are two additional subfolders: train and val. These subfolders store the defects data for training and validating the specified DL-based models respectively. The information contained in the label data mainly includes data type, number of labels and label coordinates. The files within the labels subfolder comprise details such as the file name, label category, center coordinates of the defective object’s bounding box, as well as the width and height of the defective object’s bounding box. These measurements are normalized, representing proportions relative to the image’s width and height. The label file has the capability to encompass multiple defective objects, with each object delineated on an individual line.

The folder Data_COCO comprises three main subfolders: train2017 for the training set images, val2017 for the validation set images, and annotations for storing label files. In the annotations subfolder, there are two .json format label files: instances_train2017 and instances_val2017 which are used to store label information in the training and validation sets, respectively. These label files contain essential information such as images, annotations and categories. The images section includes image ID, file name, width, and height. Annotations part comprises annotation ID, associated image ID, defect object category ID, bounding box coordinates (bbox), defect object area, and more. The categories section provides details on category ID and category labels.

## Technical Validation

To ensure the reliability of the DsPCBSD+ dataset for this study, a comprehensive manual examination was conducted on all images and their corresponding label annotations. This rigorous review process engaged five experts with substantial experience in the PCB manufacturing industry. These experts meticulously scrutinized each image in the dataset, carefully validating label information to identify potential omissions or inaccuracies. For situations prone to causing annotation discrepancies, such as similarities between different defect categories, a defect spanning multiple elements of PCB, or the overlap of multiple defect annotation boxes in the same region, a collective discussion involving five team members was conducted to determine the labeling categories and positions for these defects. In this collaborative discussion, a comprehensive consideration was given to factors such as the severity of each defect’s impact on PCB performance, the proportion of defects in various locations (elements), and the visibility of defects.

The DsPCBSD+ dataset offers two dataset formats, YOLO and COCO, providing convenience for utilization with the currently prominent, top-ranked DL-based detection models. To evaluate the efficacy of the curated dataset, two state-of-the-art (SOTA) models, Co-DETR^25^ and YOLOv6-L6^26^, both ranked highly in object detection on COCO, were selected for training and validation on the DsPCBSD+. The verification link for the two models can be found at https://github.com/Sense-X/Co-DETR and https://github.com/meituan/YOLOv6. The dataset was trained on computer with Ubuntu 20.04 64-bit operating system, Intel Xeon Gold 6242R processor, GeForce RTX 3090 graphics processor. The hyperparameters for the two aforementioned detection models were initially set to the recommended default values. However, to better align with the characteristics of the dataset, certain hyperparameter values such as batch size, initial learning rate, and epochs were adjusted. The dimensions of the input images have been resized to 1333 × 800 and 1280 × 1280 respecitvely for the two mdoels in the training. These modifications were made in accordance with the respective recommendations from the original research papers of each model.

**Fig. 9:**
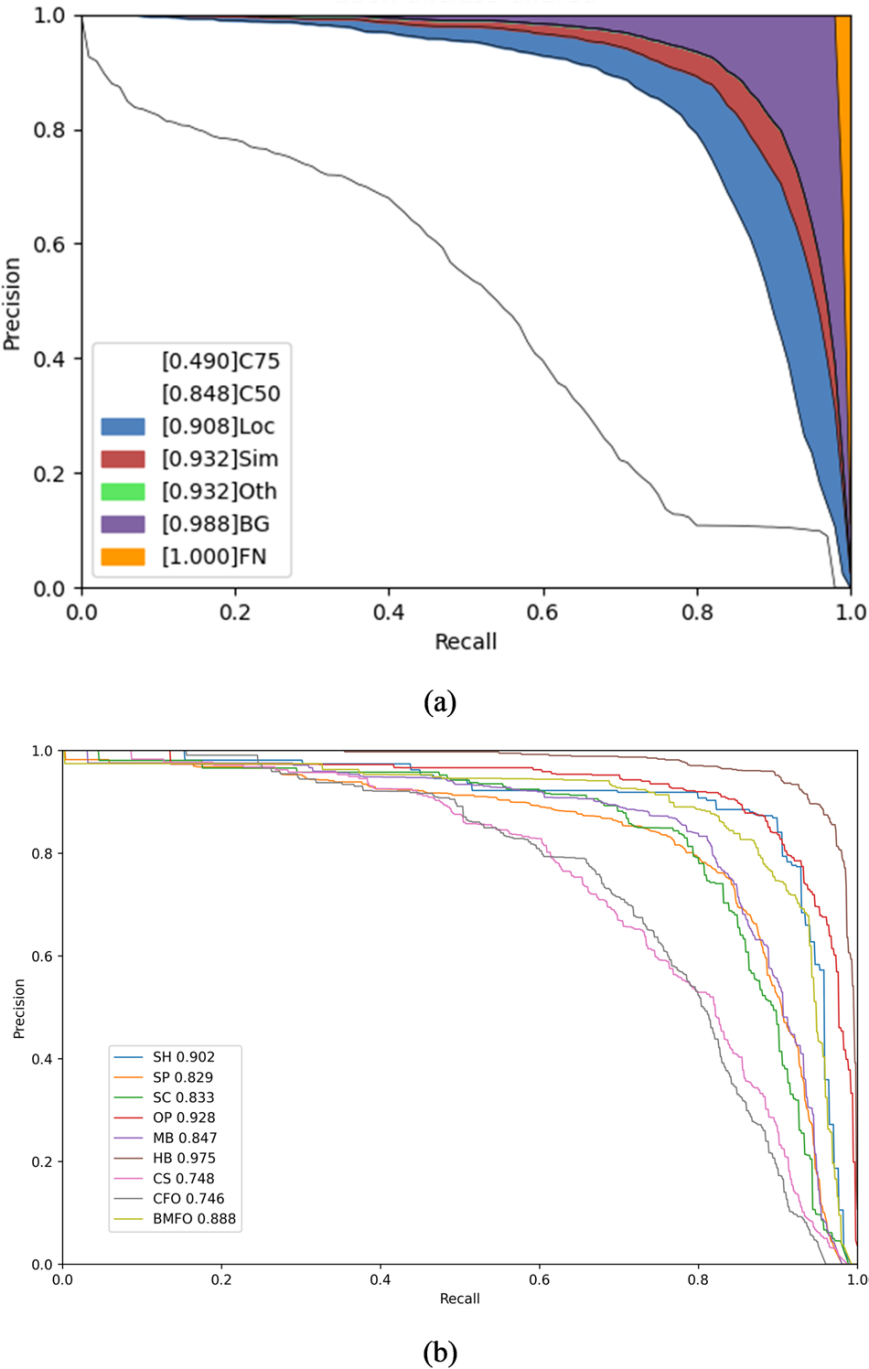
The precision-recall curves and average AP of two models (IOU = 0.50). (**a**) The result obtained by Co-DETR. C75 denotes precision at an IoU threshold greater than or equal to 0.75, while C50 represents precision at an IoU threshold greater than or equal to 0.50; Loc signifies the accuracy of the model in predicting the detected object’s position. Sim represents the model’s error in predicting the shape or appearance similarity of the object. Oth encompasses errors in other aspects that do not fall under the specific types of Loc or Sim errors. BG indicates that the model erroneously predicted a background region as an object. FN represents objects that the model failed to detect. (**b**) The result obtained by YOLOv6-L6. The number following each class of defect represents the AP for that specific category.

**Fig. 10:**
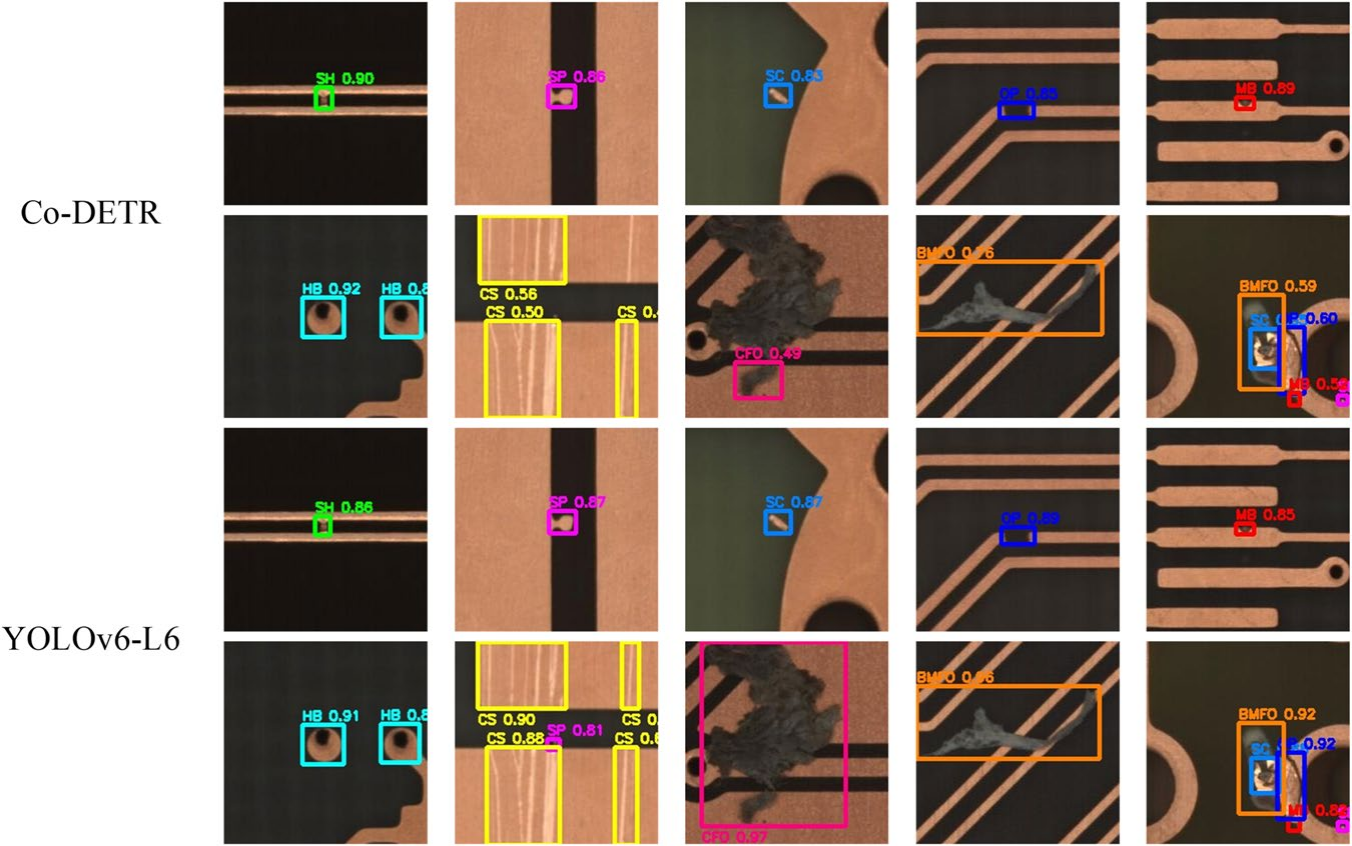
Instances of detection result obtained by Co-DETR and YOLOv6-L6.

The training time for Co-DETR and YOLOv6-L6 on the test device configuration used in this study are approximately 69 minutes and 721 minutes, respectively. Figure 9 illustrates the precision-recall curves for Co-DETR and YOLOv6-L6, which are generated using their respective default tools. The benchmark results of the mean average precision (mAP) with different Intersection over Union (IoU) are summarized in Table 2, while Table 3 presents the average precision (AP), precision (P), recall (R), and F1 score for each defect category at IoU of 0.50 for the two models. Regarding the Co-DETR detection model, the mAP value for all defect categories at an IoU of 0.50 is approximately 0.848, as depicted by curve C50 in Fig. 9(a). On the other hand, for YOLOv6-L6, the overall mAP is approximately 0.851. Besides, Fig. 10 displays several instances of detection results generated by Co-DETR and YOLOv6-L6. These results validate the reliability and practicality of the DsPCBSD+ dataset, as demonstrated by the high detection performance and accuracy.

**Table 2:** The mAP results of different models in DsPCBSD+.

Model	AP_50_	AP_75_	AP_50:95_	AP_S_	AP_M_	AP_L_	AR_50:95_	AR_S_	AR_M_	AR_L_
Co-DETR	0.848	0.490	0.492	0.425	0.554	0.671	0.668	0.600	0.743	0.846
YOLOv6-L6	0.851	0.525	0.514	0.405	0.597	0.681	0.654	0.590	0.748	0.812

AP_50_, AP_75_, and AP_50:95_ respectively represent the AP at IoU thresholds of 0.50, 0.75, and within the range from 0.50 to 0.95. AP_S_, AP_M_, and AP_L_ respectively denote the AP for small, medium, and large objects at IoU thresholds from 0.50 to 0.95. AR_50:95_, AR_S_, AR_M_, and AR_L_ represent the Average Recall at IoU thresholds from 0.50 to 0.95, for total, small, medium, and large objects.

**Table 3:** The benchmark results of each defect category for the two models in DsPCBSD+.

Model	Metric	SH	SP	SC	OP	MB	HB	CS	CFO	BMFO
Co-DETR	AP	0.885	0.842	0.825	0.898	0.836	0.973	0.727	0.741	0.900
	P	0.838	0.746	0.750	0.860	0.793	0.866	0.688	0.734	0.836
	R	0.824	0.824	0.736	0.819	0.783	0.980	0.761	0.659	0.854
	F1	0.831	0.783	0.743	0.839	0.788	0.920	0.723	0.695	0.845
YOLOv6-L6	AP	0.898	0.826	0.830	0.923	0.843	0.971	0.746	0.743	0.881
	P	0.649	0.676	0.627	0.677	0.644	0.726	0.442	0.547	0.687
	R	0.876	0.883	0.804	0.911	0.846	0.987	0.853	0.697	0.902
	F1	0.746	0.766	0.705	0.777	0.732	0.836	0.582	0.613	0.780

From Table 2, it can be observed that as the detected defect objects become smaller, both Co-DETR and YOLOv6-L6 show lower average detection precision (AP_S_, AP_M_, and AP_L_) and recall (AR_S_, AR_M_, and AR_L_). The challenges inherent in detecting small defects often involve constraints such as limited visual features, low resolution, and potential occlusion by surrounding elements. Given the increasing complexity of PCBs with a proliferation of tiny and intricate details, the corresponding defects have become finer and smaller. Therefore, models tailored for the detection of these small defects must adeptly navigate these challenges with sensitivity, capturing intricate details to ensure precise detection.

**Fig. 11:**
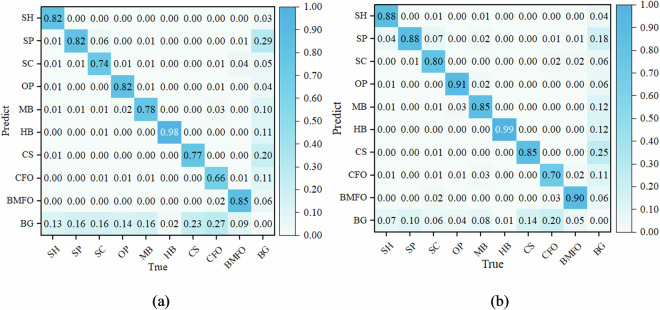
The normalized confusion matrix of the two models. (**a**) Co-DETR. (**b**) YOLOv6-L6. In the normalized confusion matrix, rows represent the predicted categories, while columns represent the true categories. Diagonal cells indicate accurately predicted labels. Each number in a cell represents the proportion of the model predicting the true category as the corresponding rows category.

**Fig. 12:**
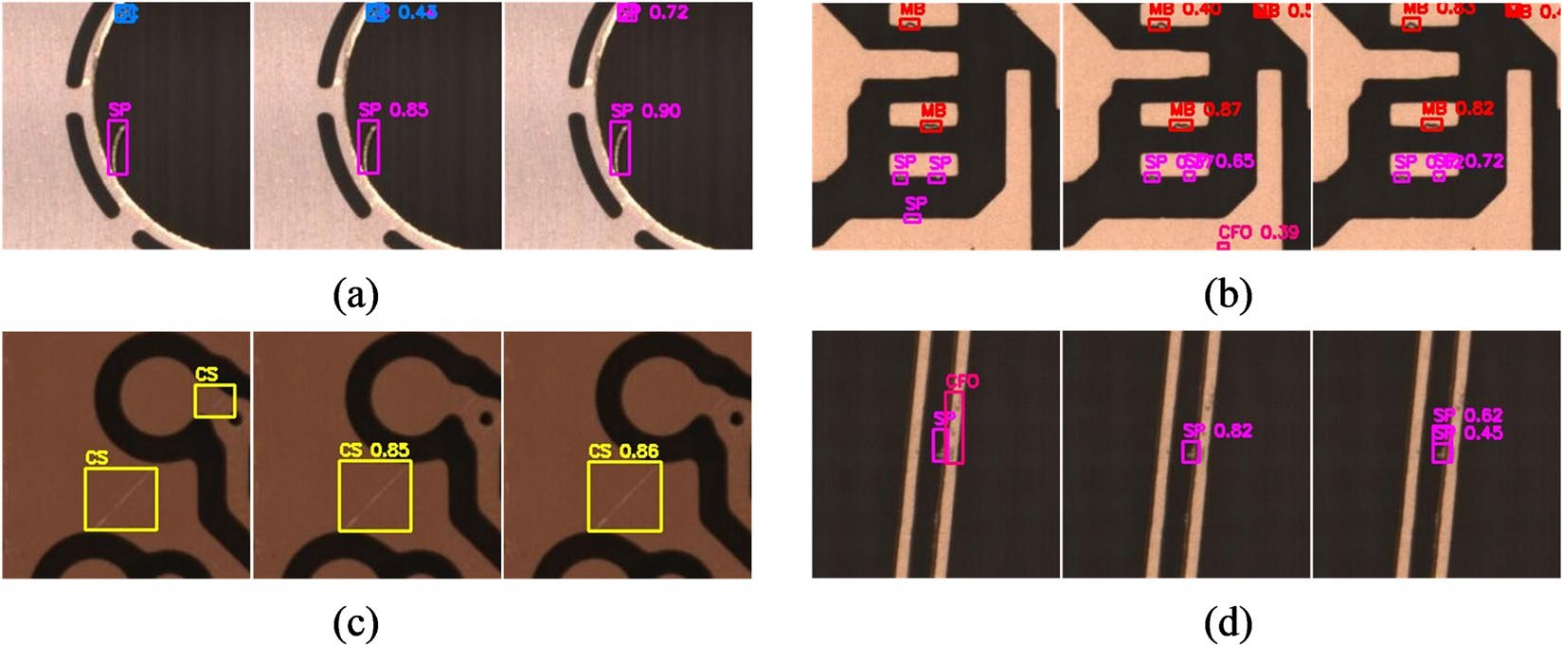
The examples of misidentification or missed detection. (**a**) SC misidentified as SP; (**b**) Missed detection of SP; (**c**) Missed detection of CS; (**d**) Missed detection of CFO. The first column in each subfigure presents the annotated instance, while the second and third columns display the detection results obtained by Co-DETR and YOLOv6-L6, respectively.

From Table 3, it is evident that both Co-DETR and YOLOv6-L6 demonstrate relatively high AP and R across various categories of defects. Figure 11 illustrates the confusion matrix for each defect category, including the background (BG), for both models. Based on the Table 3 and the confusion matrix in Fig. 11, it can be observed that the proportion of each type of defect being misclassified as other defect categories is relatively low, with only SC being misclassified as SP at a relatively higher rate (0.06 for Co-DETR and 0.07 for YOLOv6-L6). This could be because when SC is closely adjacent to the conductor, its features are similar to SP, making it prone to misclassification as SP. At the same time, it can be seen that both Co-DETR and YOLOv6-L6 exhibit relatively high rates of missing SP, CS, and CFO defects. From the perspective of defect features, the majority of SP defects are small in size, rendering them relatively inconspicuous. Conversely, CS defects exhibit significant internal size variations, making smaller scratches particularly prone to being overlooked, as evidenced by a CS defect that escaped detection by Co-DETR, as depicted in the second row and second column of Fig. 10. CFO defects display considerable disparities in color, size, and morphology, with some foreign objects being similar in color to the background. Consequently, CFO defects are susceptible to being erroneously identified as background, echoing the CFO defect that evaded detection by Co-DETR in the second row and three column of Fig. 10. The additional instances of misidentification or missed detection mentioned above are further depicted in Fig. 12.

To better validate the robustness and reliability of the dataset, additional experiments were conducted using five-fold cross-validation. Based on the initial dataset partitioning, the training set underwent further random division into four equal segments. These segments were then merged with the original validation set, resulting in the creation of five distinct sets. Each set was cyclically utilized as a validation set once, while the remaining four sets served as training sets. Consequently, these sets not only represent the original dataset but also variations where each segment of the training set took turns as the validation set. Five-fold cross-validation experiments were carried out for each of the two models (represented respectively by Co-DETR_5_ and YOLOv6-L6_5_), and the resulting benchmark outcomes are detailed in Table 4. It can be observed that the performance metrics of Co-DETR_5_ and YOLOv6-L6_5_ exhibit minimal deviation from the results obtained with the original dataset partitioning as shown in Table 2. It can be inferred that the different folds of the constructed DsPCBSD+ contain a sufficient number of representative samples, and the variations in the partitioning have minimal impact on the model results, demonstrating that the DsPCBSD+ effectively covers and represents the entire sample space.

**Table 4:** The five-fold cross-validation results of different models in DsPCBSD+.

Model	AP_50_	AP_75_	AP_50:95_	AP_S_	AP_M_	AP_L_	AR_50:95_	AR_S_	AR_M_	AR_L_
Co-DETR_5_	0.840	0.483	0.484	0.420	0.551	0.547	0.648	0.612	0.746	0.858
YOLOv6-L6_5_	0.837	0.512	0.502	0.405	0.591	0.614	0.651	0.596	0.748	0.823

## Usage Notes

Table 5 compares the defect categories in DsPCBSD+ with those in existing PCB defect datasets. Typically, most existing PCB datasets overlook defects like hole breakout, foreign objects, and scratches. However, these defects are very common on the surface of PCBs in actual production. The images collected in this study also show that hole breakout, foreign objects, and scratches account for a large proportion. Therefore, by incorporating these three types of defects into its classification, DsPCBSD+ can better cater to the practical requirements of PCB product quality inspection. Additionally, existing datasets lack detailed explanations of classification standards. Typically, various defects are only introduced through carefully selected defect image instances that are easily distinguishable. However, descriptions detailing the defect formation location, cause, and morphological characteristics are not provided. The absence of specific classification standards during the dataset creation process poses a risk of misclassification, especially for defects sharing strong inter-class similarities, such as Spur and Spurious copper found at the edge of the conductor.

**Table 5:** Comparison of defect categories in DsPCBSD+ with those in existing PCB defect datasets.

Datasets	Defects categories
DeepPCB^1^	Open, Short, Mousebite, Spur, Copper, Pin-hole
MeiweiPCB^12^	Single type of defect
PKU-Market-PCB^13^ and its extended version^14–16^	Missing hole, Mouse bite, Open circuit, Short, Spur, Spurious copper
Hu *et al*.^17^	Open circuit, Short course, Mouse bite, Spur, Pinhole, Solder ball
Liao *et al*.^18^	Bumpy or broken line, Clutter, Scratch, Line repair damage, Hole loss, Over oil-filling
Adibhatla *et al*.^19,20^	Single type of defect
Pham *et al*.^21^	
PCB-2^11^	Real defect, Pseudo defect
Li *et al*.^22^	Copper short, Short, Open, Near open, Near short
DsPCBSD+^24^	Short, Spur, Spurious copper, Open, Mouse bite, Hole breakout, Conductor scratch, Conductor foreign object, Base material foreign object

**Table 6:** The site-packages and corresponding version for the two networks.

Co-DETR		YOLOv6-L6	
Site-packages	Versions	Site-packages	Versions
Python	3.7.11	Python	3.8.18
Pytorch	1.11.0	Pytorch	1.13.1
Torchvision	0.12.0	Torchvision	0.14.1
Mmcv-full	1.5.0	Matplotlib	3.7.4
Mmdet	2.25.3	Numpy	1.23.5
Mmengine	0.10.2	Opencv-python	4.8.1.78
Numpy	1.21.6	Pillow	10.1.0
Openmim	0.3.9	Pycocotools	2.0.7
Opencv-python	4.9.0.80	Pyyaml	6.0.1
Pyyaml	6.0.1	Requests	2.31.0
Scipy	1.7.3	Scipy	1.10.1
Tqdm	4.65.2	Tqdm	4.66.1

However, the classification scheme and the dataset have their own limitations. Firstly, all the defects are limited to 2D due to the AOI’s cameras lacking 3D depth information. As a result, defects such as raised or recessed areas cannot be identified. Secondly, the images selected for DsPCBSD+ are from the inner and outer layers of boards after etching, without considering defect images after the solder mask. Thirdly, the defect images in DsPCBSD+ are all cropped images of local areas from the entire board. In practical applications, it is necessary to integrate these local images into the entire board and then mark the positions of defects on the entire board to facilitate localization by inspection personnel. These limitations should be considered when using DsPCBSD+ for practical applications.

### Supplementary information

DsPCBSD+

## Data Availability

As mentioned earlier, the DsPCBSD+ dataset is accessible on the figshare data repository^24^. Additionally, the Python code for the hash value matching method, utilized to filter highly similar images, is provided alongside the dataset and named Hash.py. Researchers can perform label format conversion from VOC format to YOLO, and YOLO format to COCO format using the resources available at the following links: https://github.com/RapidAI/VOC2YOLO, https://github.com/RapidAI/YOLO2COCO. These links offer the necessary code for label format conversion, accompanied by a README file that serves as a helpful reference. The annotation tool LabelImg is available for download on the official website at https://github.com/heartexlabs/labelImg. For dataset verification using Co-DETR and YOLOv6-L6 codes, the following website links can be visited at: https://github.com/Sense-X/Co-DETR and https://github.com/meituan/YOLOv6. The site-packages and their corresponding versions used for the aforementioned two networks are provided in Table 6. The software packages can be obtained by accessing the links specified in the README files of the respective networks and can be easily installed using the Python package installer (pip).
